# Built environment in programs to promote physical activity among Latino children and youth living in the United States and in Latin America

**DOI:** 10.1111/obr.13236

**Published:** 2021-04-06

**Authors:** Olga L. Sarmiento, María Alejandra Rubio, Abby C. King, Natalicio Serrano, Adriano Akira F. Hino, Ruth F. Hunter, Nicolas Aguilar‐Farias, Diana C. Parra, Deborah Salvo, Alejandra Jáuregui, Rebecca E. Lee, Bill Kohl

**Affiliations:** ^1^ School of Medicine Universidad de los Andes Bogotá Colombia; ^2^ Department of Epidemiology and Population Health Stanford University School Medicine Stanford California USA; ^3^ Stanford Prevention Research Center, Department of Medicine Stanford University School Medicine Stanford California USA; ^4^ Prevention Research Center Brown School at Washington University in St. Louis St. Louis Missouri USA; ^5^ Health Sciences Graduate Program, School of Medicine Pontifícia Universidade Católica do Paraná Curitiba Brazil; ^6^ Centre for Public Health, School of Medicine, Dentistry and Biomedical Sciences Queen's University Belfast Belfast UK; ^7^ Department of Physical Education, Sports and Recreation Universidad de La Frontera Temuco Chile; ^8^ Prevention Research Center Brown School at Washington University in St. Louis Scholar Institute of Public Health St. Louis Missouri USA; ^9^ Department of Physical Activity and Healthy Lifestyles, Center for Nutrition and Health Research National Institute of Public Health Cuernavaca Mexico; ^10^ Center for Health Promotion and Disease Prevention, Edson College of Nursing and Health Innovation Arizona State University Phoenix Arizona USA; ^11^ School of Public Health in Austin Department of Epidemiology Human Genetics and Environmental Sciences Michael and Susan Dell Center for Advancement of Health Living Austin Texas USA

**Keywords:** built environment, Latin American youth, Latino children and adolescents, physical activity

## Abstract

To prevent obesity among Latino youth in the United States and Latin America, it is necessary to understand the specific context and interplay of physical activity (PA) and the built environment (BE). This paper aims to advance the research agenda of BE and PA for obesity prevention in Latin America and among Latino youth in the United States by (1) identifying environmental indicators to inform the design of interventions and policy, (2) identifying interdisciplinary methodological approaches for the study of the complex association between BE and PA, and (3) presenting case studies of PA‐promoting BE programs. A group of U.S. and Latin American scientists collaboratively worked to propose innovative indicators of the BE, methodological approaches for the study of the complex association between BE and PA, and review case studies of PA‐promoting BE programs in both regions. The results identified gaps in knowledge, proposed environmental indicators (e.g., landscape, street design, mobility patterns, and crime and safety), reviewed methodological approaches (social network analysis, citizen science methods), and case studies illustrating PA‐promoting BE programs (i.e., play streets, active school transport, and school setting interventions). The obesity prevention among Latino and Latin American youth requires advanced research on BE and PA addressing context‐specific priorities and exchanging lessons learned.

**Abbreviations**
ATS: active travel to school
BE: built environment
BMI: body mass index
CATCH: Coordinated Approach to Child Health
GIS: geographic information systems
GPS: global positioning system
LAC: Latin American countries
PA: physical activity
SES: socioeconomic status
SNA: social network analysis
SRTS: Safe Routes to School

## 1 INTRODUCTION

Physical activity (PA) plays a fundamental role in children and adolescents' lives, contributing to the prevention of overweight and obesity as well as cognitive, social, and emotional development and well‐being.^1^ The prevalence of physical inactivity in Latino children and adolescents living in the United States and youth in Latin American countries (LAC) is high.^2^, ^3^ We must acknowledge the importance of the cultural and built environment (BE) contexts in which PA behaviors are embedded to advance obesity research focusing on the highly diverse Latino populations living in the United States and LAC. In this paper, the BE refers to the physical environment that provides the setting for children and youth PA (i.e., schools, neighborhoods, parks, and streets).

Currently, there are over 12 million Latino youth aged 6–17 years living in the United States.^4^ This population is more likely to live in poverty and have insufficient access to trails, recreational facilities, and parks compared with other racial/ethnic minority groups.^5^ In LAC, there are over 158 million children and adolescents aged 5–19 years.^6^ This region is characterized by being one of the most urbanized, dense, unequal, and violent in the world, with over 30% of the population living in poverty.^5^ These contextual conditions provide a challenging backdrop for advancing programs promoting PA among Latino youth in the United States and youth in LAC.

It is necessary to understand the complex interplay of BE and PA to ensure supportive environments that promote PA. This requires a comprehensive interdisciplinary research focus to explore the similarities as well as differences in BE–PA associations in different cultures and regions of the Americas.^7^, ^8^

From a socioecological perspective, youth's PA is shaped by a constellation of psychological, sociocultural, family, school, and environmental factors.^9^, ^10^ Specifically, a supportive BE provides children and families with opportunities and infrastructure for free play, structured and unstructured outdoor PA, and active transport‐related behaviors.^11^ Based on this understanding, strategies have been initiated globally to promote PA behaviors among youth through interventions in three primary domains: (1) youth‐oriented, nonschool, outdoor activities in residential neighborhoods; (2) youth‐oriented, active travel patterns to and from school; and (3) school‐based interventions promoting PA.

This paper aims to contribute to advancing the research agenda of BE and PA for obesity prevention among both Latino and Latin American youth by (1) identifying environmental indicators to inform the design of location‐based interventions and policy, (2) identifying interdisciplinary methodological approaches and tools for the study of the complex association between BE and PA, and (3) presenting case studies of three types of physical activity‐promoting BE programs (i.e., play streets, active school transport, and school setting interventions).

## 2 METHODS

This paper integrates multiple data sources including the results from a workshop with experts, a systematic search of programs, and the review of specific case studies of programs featuring physical activity‐promoting BEs (i.e., play streets, active school transport, and school setting interventions) in the United States and LAC.

In November 2019, the U.S. Center for Global Health Studies of Fogarty International conducted a 2‐day workshop to address the prevention of childhood obesity in Latin America and among Latino populations in the United States. The workshop brought together U.S. and Latin American scientists researching childhood obesity prevention to share research results and lessons learned and identify common research questions.^12^ Based on the expert group consensus, we defined specific indicators that should be documented when considering BE and PA among Latino and Latin American youth: urban form and landscape, school built environment, parks and green spaces accessibility, mobility patterns, crime and safety, and children's perspectives on affordances—always acknowledging the context of socioeconomic inequalities in the United States and LAC.

The experts also underscored the importance of a complex methodological approach using interdisciplinary mixed methods, such as social network analysis and citizen science, to advance BE and PA interventions among youth. These approaches can lead to a clearer understanding of the interactions between activity‐enhancing or activity‐limiting places and PA behaviors, and its cultural‐related aspects.^9^ PA levels are affected by social norms and context‐specific factors (e.g., gender norms, safety perceptions, parental rules, and socioeconomic disparities), making it critical to advance research at the intersection of infrastructure (i.e., the physical built environment aspects) and agents (i.e., the people and communities using and interacting in the BE).^13^

We reviewed published systematic reviews and conducted an expert consultation with members of the Network of Ciclovía of the Americas and program coordinators for the availability of programs that have been implemented in the United States and LAC to promote PA among youth in the three primary domains: (1) nonschool outdoor activities (play streets), (2) active travel patterns to and from school, and (3) school setting interventions. Applying the socioecological perspective, we identified at each level (individual, interpersonal, and community) the intervention targets and aspects to further evaluate (Table 1). We identified one case study per type of program and region and conducted in‐depth interviews with academic researchers and practitioners who have been part of the reviewed case studies.

**TABLE 1:** Built environment interventions promoting physical activity among Latino and Latin American youth

	Intervention target				Evaluation instruments
Interventions	Individual level	Interpersonal level	Community level	Policy	Aspects to address
Play streets	*Physical activity intensity levels* *Motivation* • Motivation to engage in structured and unstructured PA • Preferred playing materials *Food choices and eating behaviors* *Electronic media use* *Citizen advocacy*	*Social norms shaping gender roles in play* *Families and households* • Family cohesion • Family support • Intergenerational influences	*Built environment* • Safety • Traffic *Sociocultural environment* • Social cohesion • Food environment • Sociocultural meanings of play • Civic engagement *Participating institutions*	*Government programs*	*GIS and observation instruments* • Urban form factors • Traffic data • Crime *Social Network Analysis* • Friendship networks and PA levels • What is the network supporting the continuity of the program*?* *Our Voice citizen science* • Advocacy training program for participating families to ensure sustainability and community “ownership”.
Active travel to school	*Physical activity intensity levels* *Motivation* *Confidence* *Skills* *Citizen advocacy*	*Social norms related to transport and youths' independent mobility* *Families and households* • Family support • Gender role differentiation • Intergenerational influences • Parental concerns and rules • Perceived neighborhood environment (parents and children) • Household transport choices	*Built environment* • Traffic safety • Infrastructure *Sociocultural environment* • Food environment • Civic engagement • Sociocultural meanings of ATS *Participating institutions*	*Government programs*	*GIS and observation instruments* • Urban form factors • Traffic data • Crime *Social Network Analysis* • Friend influence on ATS • Social norms related to transport *Our Voice citizen science* • Facilitators and barriers to ATS • Neighborhood walkability, school's facilities, food access, transport access, personal safety
School setting interventions	*Physical activity intensity levels* *Motivation to engage in structured and unstructured PA* *Food choices and eating behaviors* *Electronic media use Citizen advocacy*	*Social norms shaping the way youth use activity settings according to gender, age and cultural background* *Social support* *Social capital*	*Built environment* • PA resources • School facilities *Sociocultural environment* • Social cohesion • Food environment *Participating institutions*	*Government programs*	*GIS and observation instruments* • How does urban form connect school environment with other urban settings used by youth*?* *Social Network Analysis* • Friend influence on PA and food choices *Our Voice citizen science* • Co‐creation of interventions • Facilitators and barriers to PA • Citizen engagement

Abbreviations: ATS, active travel to school; GIS, geographic information systems; PA, physical activity.

## 3 RESULTS

### 3.1 BE and PA among Latino and Latin American youth

Even with considerable evidence on the associations between BE and PA from the last two decades,^14^, ^15^, ^16^ mainly coming from high‐income countries, there is limited evidence from Latino U.S. youth^10^, ^15^, ^16^, ^17^ and from LAC.^15^, ^16^ The common themes emerging from this line of research include (i) perceived access to recreational opportunities for PA in one's neighborhood or community and (ii) the importance of PA at or en route to school.^16^

With respect to recreational PA opportunities, geographical areas in the United States with predominantly Latino populations have shown a lower probability of having parks and recreational facilities.^18^, ^19^ Parents of Latino children have consistently reported limited availability of parks, facilities for PA, and clean, safe places as barriers to their children's PA.^18^, ^20^, ^21^, ^22^ Importantly, perceived neighborhood access to parks, playgrounds, and gyms typically has shown a stronger effect on promoting PA among Latino youth than objective measures of park access.^23^ This suggest the relevance of addressing both availability and perceptions of BE features to influence active behaviors among Latino youth.

Similarly, the limited evidence from LAC shows that parents' perceptions of neighborhood BE attributes are associated with children's use of parks and unstructured open spaces for PA.^24^ Regarding objective BE data, in Mexico, sidewalk availability was positively associated with PA behaviors among youth,^25^ while in Brazil, mix land use (i.e., a range of land uses including residential, commercial, and industrial to be co‐located in an integrated way to support PA), recreational facilities or venues along the route (e.g., parks), and residential density were factors associated with youth PA.^26^ Additionally, evidence from Mexico revealed that participation in school‐based sports and organized PA was higher in children living in unsafe neighborhoods and with more path obstructions, whereas the participation in unstructured types of outdoor PA was higher in neighborhoods with more pedestrian amenities, greater cleanliness, and low traffic volume.^27^ This evidence regarding the relationship between the safety conditions of the neighborhoods and unstructured outdoor play suggests similarities in the United States and LAC regarding parents' perceived BE availability and context‐specific differences regarding BE features and PA among Latino youth.

With respect to youth PA occurring en route to or at school, active commuting to school or unstructured outdoor PA in the school setting^25^, ^27^, ^28^ have been associated with school surroundings, including speed limits, crossing guards or other intersection crossing aids, and the presence of sidewalks in both the United States and LAC. Unfortunately, in the United States, there has been a rapid decline in the rates of active travel to school over the past five decades, with a decrease nationally from a 40.7% prevalence in 1969 to 10.7% in 2017.^29^, ^30^ Importantly, Latino youth are more likely to bike or walk to school compared with their White or Black youth counterparts.^31^

Evidence from LAC highlights that this behavior constitutes a major source of PA among youth, with considerably higher prevalence overall in LAC, ranging from 23.0% to 70.8%, although substantial variations by country have been reported.^26^, ^32^, ^33^, ^34^, ^35^, ^36^ Most LAC studies on the correlates of active travel to school support that in this region, this is a necessity‐driven mobility behavior (i.e., high socioeconomic status and having the means to use a private vehicle are inversely associated with such active travel). In terms of BE features, no consistent characteristic has been associated with active travel to school across the LAC region. Additionally, associations between BE and active travel to school have not been explored for public and private schools.

Underscoring the importance of the social environment when studying the BE, evidence from Mexico showed that youth with more neighborhood social ties (defined as interactions among residents) reported more PA during the week, and the number of social ties had a stronger positive association with PA than perceived neighborhood safety.^23^ In fact, studies exploring safety, crime, with PA in youth in Argentina,^24^ Brazil,^37^ Colombia,^37^ and Mexico^25^, ^38^ have shown inconsistent associations.^39^ Meanwhile, concerns about crime, gangs, and unsafe neighborhoods have emerged as barriers to PA frequently reported among children of Latino parents living in the United States.^40^, ^41^, ^42^

Together, the limited research on BE and PA among Latino U.S. youth and LAC youth reveals the need for further research to better define what “activity‐friendly” environments actually mean in these different regions.

### 3.2 Built environment indicators affecting PA behaviors

The expert group underscored the need to develop indicators within the following topics to advance the understanding of BE and PA among Latino and Latin American youth: urban landscape and street design, parks and green areas, mobility patterns and activity places, crime and safety, and children's perspectives on affordances.

#### 3.2.1 Urban landscape and street design

The spatial configuration and composition of urban environments can affect PA behaviors. As cities where Latino and Latin American youth live continue to expand and densify, quantifying their spatial configuration and accurately projecting their future dynamics becomes critical for PA and obesity prevention. Within the spatial configuration, the urban landscape domain measures how urban development is configured within each city and includes variables like fragmentation, isolation, shape of developed urban areas, and city density that could provide proxy measurements of compact cities versus urban sprawl.^43^ The street design domain typically includes street connectivity, street length, and directness, which can provide proxy walkability measurements. Advances in geospatial analyses and remote sensing offer a unique opportunity for comparable urban landscape and street design metrics.^43^, ^44^ These innovative metrics can be calculated using urban footprint data from the Global Urban Footprint project and OpenStreetMaps. In turn, these metrics could be used to explain, in part, PA and mobility patterns of Latino and Latin American youth, leading to more inclusive urban planning policies. There are current efforts to develop these indicators in the United States and in LAC^43^ where standardized geographic scales have been applied and could be linked to survey data. However, to our knowledge, there are no studies on the association of these indicators and PA among Latino youth.

#### 3.2.2 Parks and green areas

Parks are vital components of communities, offering opportunities for groups, families, and individuals to enjoy outdoor activities together and alone, including PA. Parks and green spaces are measured on dimensions of quantity, the absolute number or space devoted to them, and the quality of the features and amenities of the park.^45^, ^46^ Also, the physical characteristics of parks and other greenspaces are defined as elements that people use for PA, such as sport or recreation fields, trails, or courts.^47^ Features may also include shared areas including plazas or pavilions that might be used for civic events or to host activities, such as dancing or group exercise classes.^47^ Other physical elements include park amenities, such as water fountains, restrooms, lighting, or benches.^47^ Having more features and better amenities is usually associated with more PA, particularly when parks or greenspaces are well maintained and safe.^47^ Parks with a large number of incivilities such as broken glass, trash, tagging/graffiti or other nuisances can discourage their use for physical activities and may be perceived as unsafe.^47^ Of note, systematic differences in neighborhood park quantity and quality along ethnic or socioeconomic status lines have not been consistently observed in the United States and may depend less on socioeconomic differences and more on local policy or civic involvement.^48^

Recent research on green space has used a satellite‐based measure of green space, the normalized difference vegetation index, which is a proxy measure of neighborhood greenness or availability of green space.^43^ Key benefits of this index have been its simplicity to understand, and reliability in capturing vegetation levels in any parts of the world where satellite data are available, but these studies have not included children. The use of park‐based indicators and green areas where Latino and Latin American youth live could provide a better understanding of the complex relationships between such places and PA.

#### 3.2.3 Mobility patterns and activity places

Most studies examining mobility patterns and activity places in Latino and Latin American youth rely on self‐report survey data that may help identify individual facilitators or barriers to PA.^49^, ^50^ However, such self‐reported survey data may not be as useful in identifying multilevel or ecological influences of PA. Innovative strategies that examine behavioral patterns and routes of children's daily activities may help in understanding and in the identification of their activity places. Location‐based data, including global positioning system (GPS) devices and geographic information systems (GIS) data, have proven useful in examining PA in other populations.^51^, ^52^, ^53^ However, the assessment of children's activity places using location‐based data is limited among Latino youth.^54^, ^55^ The use of such technologies to explore multilevel influences of PA will provide a better understanding of mobility patterns and activity places in Latino and Latin American youth.

#### 3.2.4 Crime and perceived safety

Crime and safety‐related factors of neighborhood environments strongly shape parents' restriction of their child's PA and independent mobility. However, most studies in this area have used isolated questions about the perception of safety or crimes and thus may not adequately capture this construct. Additionally, social and individual factors are potential moderators between perceived and objective measures of neighborhood safety, such as community cohesion, gender (of parent and child), child's age, education, and acculturation.^56^ Perceived crime and traffic safety are typically measured using surveys aimed at reporting signs of physical and social disorder, stranger danger, and perceived levels of traffic hazards.^56^ Meanwhile, objective measures of crime and traffic safety are assessed using in‐person, context‐specific observational audits, GIS, or available crime and safety databases.^56^ It is important to use ecologic approaches that combine perceived and objective measures of neighborhood safety to capture more comprehensively factors at the individual, family/parent, and neighborhood levels.

#### 3.2.5 Affordances

Within a functional approach seeking to maximize BE potential to increase PA and outdoor play among youth, the concept of affordances becomes relevant. In the context of play, affordances are the functionally significant properties of the environment that satisfy children's needs, interests, motivations, or capabilities (i.e., flat surface and climbable elements).^57^ The concept of affordances highlights the value of a variety of materials, colors, and textures for stimulating diverse interests and abilities among youth. Considering affordances is a way to think about the relations between spatial features and children's behavior^58^ while examining the appropriateness of BE interventions. It can be a useful concept for advancing research, particularly to include children's perceptions of the BE, which can facilitate opportunities to co‐create appropriate interventions, involving policymakers, researchers, and youth, the direct users.

### 3.3 Interdisciplinary methodological approaches

#### 3.3.1 Social network analysis: A tool to integrate social environment constructs into built environment research aimed at physical activity

**FIGURE 1:**
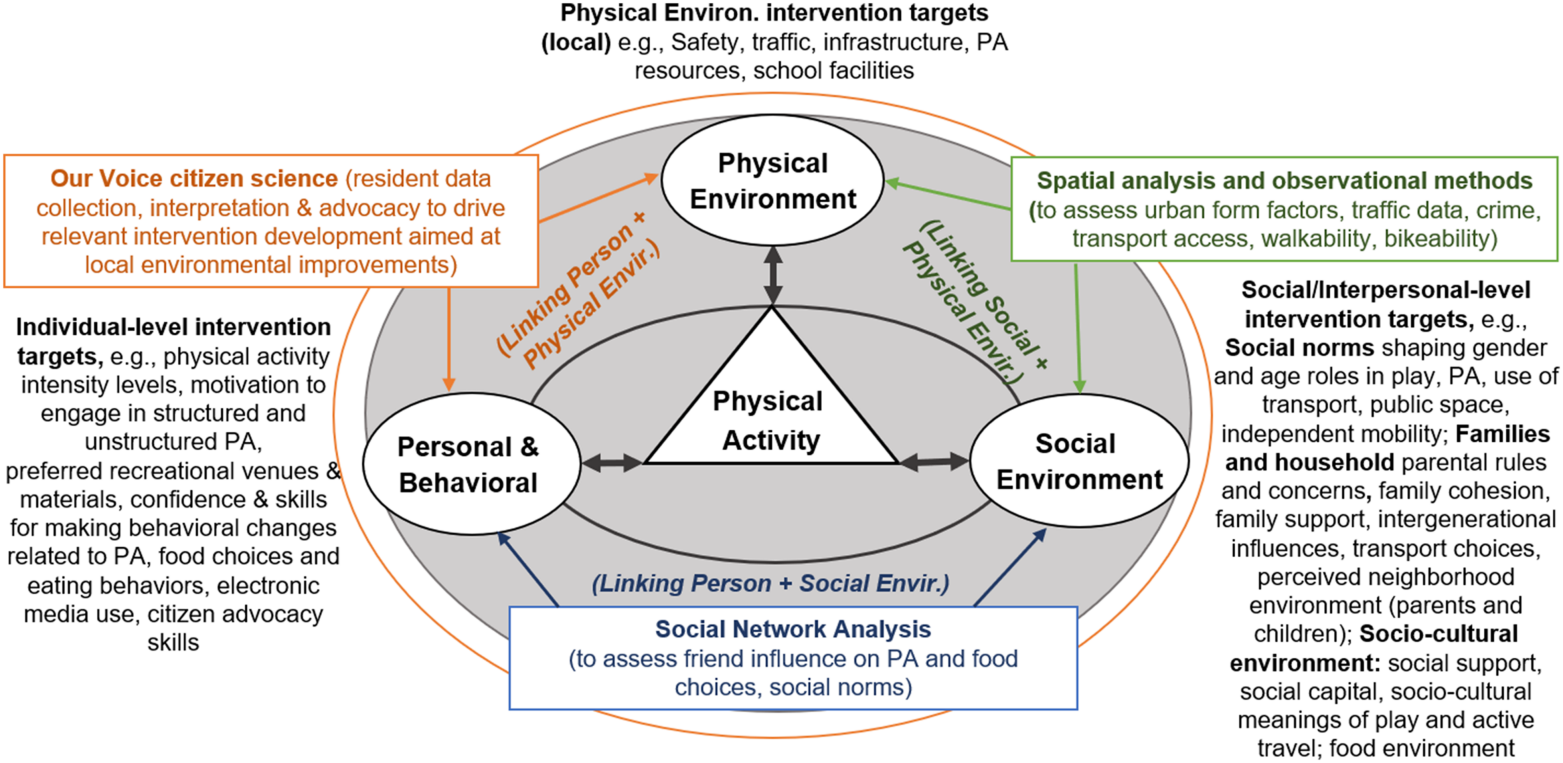
Methodological approaches to evaluate intervention targets of built environment interventions promoting physical activity among youth. Adapted from King, IJBNPA^60^

Understanding and using inherent social structures of children and adolescents may provide a cost‐effective way of encouraging PA behaviors and better utilizing the BE in which youth play, travel, and study.^59^ BE settings such as schools, neighborhoods, parks, and streets are “social settings” with inherent social constructs that influence PA behavior.^8^ However, community social networks (i.e., the people around us) and norms (i.e., the accepted and appealing standards of behavior in a social group) are rarely integrated into our PA interventions^7^ (Figure 1).

Socially activated interventions purposefully utilize the social structures in which individuals are embedded, such as social networks and norms, as drivers to support PA behaviors.^61^, ^62^, ^63^ Tools such as social network analysis (SNA) and game theory can help us better understand the social environment and how we might best integrate influential social constructs in BE–PA interventions.^64^, ^65^, ^66^ Such tools can be instrumental to assess the mechanisms by which social structures influence PA behaviors. For example, we can assess the mechanisms by which social norms affect the promotion of PA among female and male Latino youth after the implementation of park programs. They may also facilitate the development of more powerful interventions aimed at addressing the obesity epidemic among youth in both regions through, for instance, capitalizing on social ties and the role of friends in influencing PA levels as well as other relevant health behaviors.^64^, ^65^, ^66^

SNA has been used to assess potential mechanisms through which PA interventions in school settings accomplish behavior change. One study in the United States has used SNA to examine the associations between individual health indicators (PA and BMI) and friendship groups among adolescents. The results revealed evidence of friend influence on BMI and PA in two public schools, one predominantly white and the other ethnically diverse (40.2% Latino students).^67^ Another U.S. study, using accelerometer data, SNA, and agent‐based modeling, examined how two afterschool interventions could leverage social networks to increase PA in children.^68^ The intervention that targeted student opinion leaders effectively increased the average level of PA across the entire network. In contrast, the intervention that targeted the most sedentary children was the best at increasing their PA levels. The findings indicate that implementing different types of interventions may depend on whether the goal is to shift the entire distribution of PA or to influence those most adversely affected by inactivity.^68^

In Colombia, SNA was used to assess the potential effect of social cohesion of a PA school‐based intervention during recess implemented on school facilities.^69^ The program potentiated with text messages to encourage children to participate in school‐based recreational programs had a higher positive impact on the creation of friendships and social cohesion than the program alone, suggesting that school‐based interventions combined with information and communication technologies might encourage social cohesion among children to modify health‐related behaviors.^69^

#### 3.3.2 The Our Voice model: Engaging youth and families as citizen scientists to build supportive environments for PA promotion

The *Our Voice* model is a community‐engaged citizen science approach that, through the use of a technology‐driven participatory process, allows “real‐time” evaluation of local built and social environmental factors influencing PA among youth and their families.^70^, ^71^ The citizen science approach can be defined generally as engaging members of the public in research processes, such as data collection, analysis, and dissemination, to contribute to scientific advancements.^70^ Youth use the Stanford Discovery Tool mobile app to collect data, via geo‐coded photographs and audio‐ or text‐based narratives and route mapping, of features of their local environments that help or hinder their PA.^72^ Such features may include neighborhood walkability, adequacy of school facilities for PA, or local elements that impact feelings of personal safety or active transport.^73^, ^74^ Youth then meet in a facilitated process, either in‐person or through a remote, web‐based meeting platform, to share their data and build consensus around high priority issues and potential solutions. The Our Voice process has also been found to strengthen individuals' personal and collective efficacy for changing behaviors and their local environments and policies.^70^, ^75^, ^76^

In North America, the Our Voice model has been used in disadvantaged communities with large proportions of Latino youth and adults. In multigenerational projects in the United States and Mexico, youth have worked in tandem with their adult family members to identify aspects of their local neighborhoods that hinder or enhance walkability and have been able to identify relevant solutions for promoting neighborhood safety for walking.^74^, ^77^ The model has also been employed in a less dense area of Santa Clara County, CA, with a significant proportion of Latino families to strengthen safe routes to school programs delivered by the County Public Health Department.^73^

In Latin America, Our Voice has been used to promote healthier schools, and local park and open street environments in urban and rural communities in Colombia.^78^ In urban schools in Bogotá, which included elementary through high‐school youth, students identified high‐priority issues related to their school environments and proposed improvements to the local school management board, such as bicycle parking area expansion, affordable healthy food offerings, and availability of drinking water dispensers.^79^ Similarly, in a school located in Barú, a deprived rural community in Colombia, students prioritized issues related to both the school and local neighborhood environments that impacted health and active living. Their advocacy process with the school administration and other decision makers contributed to identifying pollution as a barrier to health in the school and local environments, with authorities agreeing to review possible solutions for containing local the sewage, and students committing to taking better care of the general infrastructure at the school.^80^

### 3.4 Programs promoting activity places and physical activity behaviors among Latino and Latin American youth

Several initiatives in the United States and LAC have implemented BE interventions to promote PA among youth. We reviewed programs that target BE features related with the topics underscored above. Play streets foster outdoor play by temporarily limiting automobile traffic on residential streets so children and their families can safely play and engage with their community.^81^ Play streets are generally community‐based initiatives aimed at addressing the inequality in opportunities for outdoor play for children from lower SES families that may not have a park, green space, or safe shared play spaces in walking distance to their home.^82^, ^83^ Active travel to school programs are aimed at promoting playful, safe active commuting to school by targeting the context‐specific social and BE deterrents. As children are the most vulnerable pedestrians and bicyclists, active travel to school programs focus on safety, infrastructure, and education aspects to foster independent mobility, civic engagement, and environmental awareness among youth, families, and communities. Likewise, acknowledging the school is a crucial place for health promotion,^84^ the multicomponent school‐based programs are particularly useful in promoting PA as well as healthier food choices^85^ by combining BE features with other strategies.

**FIGURE 2:**
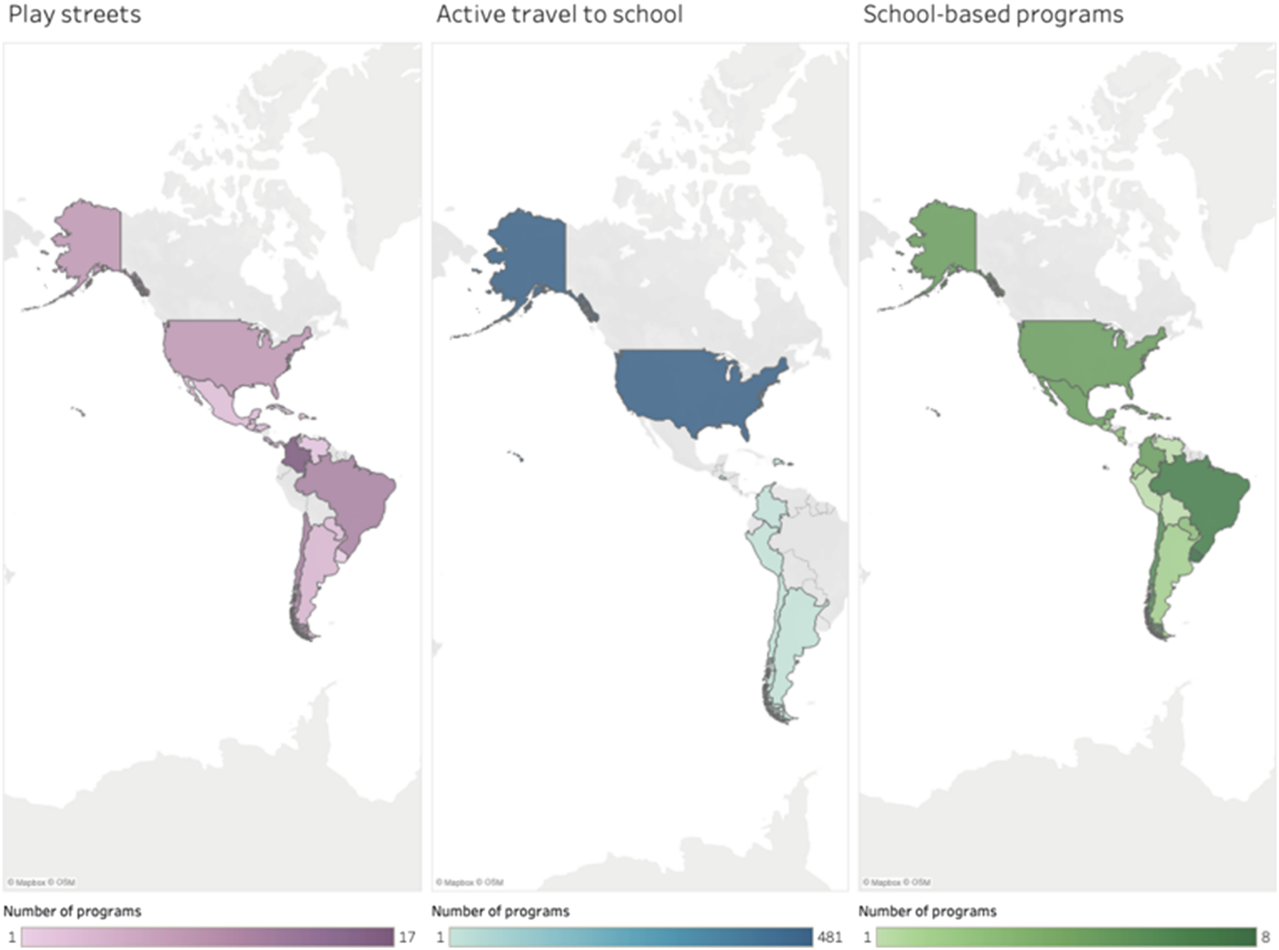
Availability of programs promoting activity places and physical activity behaviors among Latino and Latin American countries (LAC) youth

Figure 2 illustrates the availability of play streets programs, active school travel initiatives, and school setting interventions per region. We found that in the United States, the active school transit programs are more prevalent than in LAC. In contrast, play streets programs are more prevalent in LAC. Despite having found some examples, information regarding school setting programs was generally very limited. Table 2 presents the reviewed case studies.

**TABLE 2:** Case studies of built environment interventions for promoting physical activity among Latino and Latin American youth

Type of intervention	Program	Characteristics	Lessons learned
Play streets are community‐based initiatives where residents work together to temporarily limit automobile traffic on a residential street so children and their families can safely play, socialize, and actively engage with their community.^81^ They are aimed at addressing the inequality in opportunities for outdoor play for children from lower SES families that may not have a park, green space or safe shared play spaces in walking distance to their home.^82^, ^83^ Play streets differ in area size, schedule, number of participants, and type of activities depending on the decisions of the residents on and around a specific neighborhood block.	**PlayFamilias**: Latino families activating streets in Miami, Florida	Initiative promoted by partnered non‐profit organizations engaged in the empowerment of communities.^86^ It was implemented in 2017 to address the Little Havana neighborhood families' lack of safety to walk, ride bikes, or play outdoor games.	‐Play streets is a BE context‐specific malleable initiative that is adaptable to different sociocultural perspectives on play with the potential to foster intergenerational interactions and transform sedentary behaviors in parents, grandparents, and children. ‐The sustainability of play streets requires a family‐based approach to engage local residents in outdoor play. As an initiative relying on self‐organized residents of local streets, the implementation of play streets requires tailoring processes to raise awareness among parents and local residents regarding the ultimate goals of this program. ‐As low cost, easy to implement program, play streets are ideal for communities with limited green or public spaces and its contributions to a wide range of outcomes related to social and emotional well‐being should be studied further.
	**Juega en tu Barrio**: Chilean intervention for outdoor play	Research intervention conducted in 2014 in Santiago de Chile, the capital city of Chile. It has been scaled up as a program funded by the Health Ministry. In the COVID19 pandemic it has received particular attention for reducing inequities in access to open areas in neighborhoods. The intervention and non‐play street control neighborhoods (total *N* = 2) were low‐to‐middle income neighborhoods with poor urban infrastructure and high traffic/stranger danger perceptions, presenting similar crime levels.^83^ The intervention targeted all levels of the socioecological model and its results indicated that it was effective in increasing outside play and PA in children.^83^ Fifty‐three percent of children participated in more than 70% of the sessions, with more girls participating. In addition, parental safety concerns regarding outdoor play improved, as well as social cohesion among neighbors.^83^	
Active travel to school (ATS) are programs fostering the joy of safe walking and biking. They often comprise multilevel socioecological actions, including both educational activities and BE changes as strategies to promote travel behavior change, including infrastructure improvements, safety education and enforcement, and incentives for participation. Household SES and car ownership determine transportation options. In the United States, 90% of adults report using a private car to work, whereas in LAC it is 22%.^87^ Children from low‐to‐middle income neighborhoods in LAC may not have the choice to access motorized travel to school and often are exposed to high traffic risks when walking or biking to and from school.^88^	**Safe Routes to School in the United States**: Government‐supported local engagement to active school travel	The Safe Routes to School (SRTS) program was established by the U.S. federal legislation in 2005 to encourage states to develop local programs that enable more children to use ATS safely.^8^ This federal transportation program provides funding to local projects supporting safe options for walking/biking to school through improvements of the BE and noninfrastructure activities (e.g., media campaigns, police patrols, pedometer programs, bicycle equipment and rodeos, traffic safety education). SRTS programs have achieved increases in moderate to vigorous PA among school‐aged children and have contributed to improved traffic safety for school‐aged pedestrians and bicyclists in areas with SRTS intervention.^89^ With attention to equity in funding, schools that received SRTS funds between 2005 and 2012 were likely to be located in dense urban environments with a higher proportion of disadvantaged and Latino students.^90^ In 2015, the *Our Voice* citizen science intervention was used to facilitate community engagement and evaluate the implementation of SRTS in two schools in Gilroy, California, a low‐density region mainly consisting of farmland and suburban areas, where 58% of the residents self‐identify as Hispanic/Latino. The addition of the Our Voice citizen science intervention to the standard safe routes to school curriculum significantly increased walking/biking to school rates across the school year in the elementary school receiving the Our Voice intervention relative to the comparison school which did not.^73^	‐Cognizant of local issues, ATS programs are generally framed in a choice model or a need‐based model. In the United States, ATS programs aim to increase ATS by addressing the issues of distance and convenience, while in LAC cities such as Bogotá, the programs are focused on preserving ATS while improving safety and infrastructure conditions. ‐To address the distance issue, it is important to develop, in both regions, safe and fun routes networks for multimodal and secure connectivity with public transport. ‐Given the important role of parents in travel mode decision making, the educational strategies to increase parents' self‐efficacy with respect to choosing the most appropriate travel modes for their children should be included in ATS interventions. This could help in shifting the social norms toward considering active transport modes the default option for commuting in many areas. ‐ATS has potential impacts on children's social and motor development. It is important to study further outcomes related to ATS' role in strengthening such social and motor development across different age groups and youth populations. ‐Promoting collaborative community‐research partnerships has been a key strategy related to successful ATS program adoption,^73^ and deserves further systematic investigation.
	**Ciempiés**, Bogotá's centipede stampede to prevent traffic injury among children	Ciempiés (Centipede) is a program that promotes playful, safe walking to school among children in low‐to‐middle income neighborhoods. It is part of Bogotá's Niños Primero (Children First) program to enhance children's mobility while focusing on safety, infrastructure, and education components. It offers safe and fun ways for children to walk together to and from school, accompanied by personnel from the Mobility Department or local community volunteers trained on playful learning. To identify potential routes, the Mobility Department analyzed neighborhoods' data for traffic safety, number of children walking to schools, and distance from households to schools. Upon contacting schools and families, they agreed and collaboratively created 20 safe and “fun” routes. Children were given reflective clothing to wear during the walks, and neighborhood collaborators were symbolically rewarded. During the first two years of Children First (2017–2019), children's deaths in transit accidents declined 64%.^88^	
School setting interventions to promote physical activity usually are embedded in multi‐component school‐based programs for health promotion.	**The Coordinated Approach to Child Health (CATCH)**, scaled up program in the United States	CATCH is a multilevel program developed in the early 1990s in the United States to enhance the school environment to promote well‐being through five modules: PA, nutrition and cafeterias, the classroom, families and communities, and sun protection.^91^ The PA component includes physical education classes, active breaks during regular classes, active play during recess, and active leisure outside of the school setting. CATCH incorporates both inter‐personal and teacher‐led strategies with informational (campaigns) and BE (enhanced equipment in appropriate facilities) approaches. It has shown to be effective in increasing school‐based PA and preventing unhealthy weight gain.^91^, ^92^, ^93^ Over the course of 20 years, CATCH expanded from a controlled multi‐site effectiveness trial to researcher‐led translation trials, achieving institutionalization in several US cities and states,^93^ and inspiring school‐based programs in countries like Colombia and Chile. CATCH has been adapted for and scaled‐up in U.S. cities and states with a proportion of Latino youth (e.g., Texas). In addition, the CATCH program has also proven effectiveness in increasing school‐based PA in U.S./Mexico urban border communities.^92^	‐The CATCH program is a good example of successful translation, dissemination, and scale‐up of an evidence‐based PA intervention; the program has been scaled up in multiple cities and states through systems‐level institutionalization with full support at the school, health, and other government sectors levels.^93^ ‐CATCH has shown that comprehensive school‐based programs can benefit, in terms of health promotion impact, from integrating several health behavior components such as both PA and healthy eating. ‐Multi‐sectoral involvement in building an enduring culture of health in school environments requires a combination of sustained efforts which may be constrained by stakeholders' other competing priorities. It is therefore imperative that strategies be built into the multi‐sectoral process for maintaining continued attention and participation by various sectors. ‐School setting interventions often have not incorporated students' own perceptions about challenges and opportunities related to practicing healthy lifestyles in their schools. Advancing participatory‐action research with children and adolescents may enrich knowledge concerning both the content of school programs and their varied impacts on youth health, as well as setting the stage for greater involvement and engagement of students in such school programs. ‐Evaluating the impacts of school‐based programs on PA levels outside of school similarly deserve greater attention and investigation.
	**Salud Escolar**, changing school environment in Mexico	Vida Saludable and Salud Escolar,^94^, ^95^ is a new policy‐based school program launched in 2020 in Mexico by the Ministries of Health and Education with the aim of creating changes in the school to promote healthy and sustainable lifestyles among school communities, including children, teachers and parents. Salud Escolar contains three main components: (1) healthy eating, through implementing new school guidelines for foods and beverages, and prohibiting the sale of unhealthy processed and ultra‐processed foods within school canteens, (2) correct hydration, which promotes the consumption of plain water through the installation of new water fountains within elementary schools; and 3) “Suma Minutos” (“Add Minutes”, in English), which promotes PA through activity breaks, active recess, quality physical education, extracurricular activities and local and national school tournaments. The BE component comprises painting schools' playgrounds and hallways.	

Abbreviations: ATS, active travel to school; BE, built environment; CATCH, Coordinated Approach to Child Health; LAC, Latin American countries; PA, physical activity; SES, socioeconomic status; SRTS, safe routes to school.

## 4 DISCUSSION

The prevention of obesity among Latino and Latin American youth through the promotion of “activity‐friendly” environments requires to advance research on BE and PA addressing context‐specific priorities and exchanging side‐by‐side lessons learned. The collaboration among researchers from the United States and LAC allowed to underscore elements within BE and PA research and intervention fields. These included the review of existing knowledge and gaps of evidence, the potential development of evaluation indicators for BE and PA among youth, and the use of comprehensive methodological approaches (SNA, citizen science methods) to advance the implementation and research on BE interventions promoting PA.

Similarities among the United States and LAC include (1) parents' perceived limited access to recreational opportunities for youth's PA and (2) the positive association between BE features like speed limits, intersection crossing aids, and presence of sidewalks, and active travel to school among youth. In contrast, the evidence indicates differences across regions regarding (1) the influence that specific BE features may have over nonschool outdoor activities in residential neighborhoods and (2) the correlates of active travel to school. Regarding strategies that can build on the commonalities across regions to positively influence active behaviors among Latino and Latin American youth,^18^, ^19^ the most evident is to implement actions improving BE amenities (e.g., parks) and perceived access to them, targeting both youth and parents. On the other side, to fill the gap of evidence, we recommend increasing studies to determine the potential effects of urban form characteristics in PA behaviors among Latino and Latin American youth. With the decline in the rates of active travel to school in the United States, versus the high prevalence of active travel to school in LAC, finding consistent associations between BE features and active travel to school across the LAC region is of particular importance, as lessons can be learned and replicated.

Furthermore, building indicators within each of the reviewed topics (urban landscape and street design, parks and green areas, mobility patterns and activity places, crime and perceived safety, affordances) will contribute to fill the gap of evidence while comparing regions. Measuring BE features with specificity will help to inform the design of interventions enhancing safe, appealing, inclusive “activity‐friendly” environments for Latino and Latin American youth in each context. In addition, applying interdisciplinary methodological approaches such as SNA and citizen science will contribute to integrate social environment constructs into BE research and engage youth, families, and stakeholders as citizen scientists in building supportive environments for PA promotion. The use of some of these methods was illustrated within the case studies.

The reviewed U.S. and LAC case studies represent innovative programs targeting the BE to prevent or control obesity by promoting PA. The case studies have the potential to affect outdoor activities, active travel patterns, and PA in school settings. The reviewed programs typically have been developed to be malleable to context‐specific needs and local sociocultural aspects. One of the main conclusions from reviewing the case studies is that in addition to transforming the BE, these programs must impact the social structures where the Latino and Latin American youth are embedded. Play streets involve varying organizations and implementation formats^96^ and emphasize the sociocultural meanings of play to successfully engage families to reclaim the streets for PA and outdoor play. Likewise, active travel to school can be leveraged by including play and engaging the community.^73^ Despite the different possible factors shaping safety concerns limiting outdoor play (e.g., BE walkability in the United States vs. crime in Santiago), or urban form characteristics hindering active travel to school (e.g., distance in the United States vs. BE walkability and bikeability in Bogotá), the programs can create supportive environments and transform parents' and youth's perceptions toward BE features that facilitate active behaviors.

Second, we found that despite the school setting programs have been implemented and scaled‐up as policy‐based responses to the prevention and control of childhood obesity in the U.S. and LAC countries, this information was generally very limited in the LAC region. Importantly, school setting interventions have acknowledged the relevance of the interaction of PA programs and the food environments. More efforts should be encouraged to underscore that any program aiming to promote PA as a means to address childhood obesity necessarily requires to be implemented along with similar strategies trying to provide healthy food environments. Further research and intervention efforts could encourage the synergistic relations between food choices and PA for impacting health in youth, both within and outside school environments. Likewise, it is relevant extending investigations to rural areas, which have received substantially less focus in this field.

Furthermore, future research will need to combine mixed‐methods to more fully assess active behaviors among youth in advancing our understanding of the interactions between activity places and PA behaviors, as well as the culturally and socially related aspects of PA promotion (social network analysis, spatial analysis, systematic observation, and community‐engaged citizen science). Hearing the voices of youth might contribute to further implementation of gender‐specific and age‐appropriate interventions, while also empowering youth as agents of change responsible for co‐creating healthy environments. In this manner, the Our Voice model may be useful as an advocacy training program to foster improvement, sustainability, and community “ownership” of the programs. Likewise, SNA might provide useful insight concerning the extent to which social capital and norms shape active behaviors, particularly in relation to age, gender, and cultural background. Using SNA in each intervention will help to promote the understanding of who the people are who enroll in the activity and how local and external stakeholders engage and maintain the initiative.^97^ Linking a GPS tracker with SNA can help to improve understanding of which children and families practice active school travel, measure destinations other than school, and study how PA behavior spreads and may be impacted by parental rules as well as children's friends. It is relevant to include participatory methodologies that engage youth and the diversity of stakeholders involved in advancing such programs. More qualitative research is needed to evaluate youth's perception of the BE and identify their safety concerns and motivations to engage in PA. It is also beneficial to evaluate whether parental perceptions of environmental attributes are of greater importance than children's perceptions.

## 5 CONCLUSION

Advancing research on BE and PA for Latino and Latin American youth requires addressing context‐specific priorities and exchanging side‐by‐side lessons learned from United States and LAC. Sustaining and disseminating effective policies and programs in both regions will require continuously collecting contextually relevant evidence in environmental topic areas and analyzing how together they impact youth's PA. Through being cognizant of age and gender appropriateness, family and community engagement strategies, relevant social network structures, and policies aimed at intersectoral efforts to change the urban environment, notable advances in promoting PA among youth living in the United States and LAC may be more likely to occur. Such advances, in combination with the promotion of healthy diets, may further the quest to stem the tide of obesity among Latino and LAC youth.

## CONFLICT OF INTEREST

The authors declared no conflict of interest.

## References

[1] Poitras VJ, Gray CE, Borghese MM, et al. Systematic review of the relationships between objectively measured physical activity and health indicators in school‐aged children and youth. Appl Physiol Nutr Metab. 2016;41(6 [Suppl. 3]):S197‐S239. 10.1139/apnm-2015-0663 27306431

[2] Singh GK, Yu SM, Siahpush M, Kogan MD. High levels of physical inactivity and sedentary behaviors among US immigrant children and adolescents. Arch Pediatr Adolesc Med. 2008;162(8):756‐763. 10.1001/archpedi.162.8.756 18678808

[3] Evenson KR, Arredondo EM, Carnethon MR, et al. Physical activity and sedentary behavior among US Hispanic/Latino youth: the SOL youth study. Med Sci Sports Exerc. 2019;51(5):891‐899. 10.1249/MSS.0000000000001871 30570586 PMC6465089

[4] U.S. Census Bureau . Population Publications—U.S. Census Bureau. Population publications. https://www.census.gov/prod/www/population.html. Published 2019. Accessed July 27, 2020.

[5] Diez Roux AV, Slesinski SC, Alazraqui M, et al. A novel international partnership for actionable evidence on urban health in Latin America: LAC‐urban health and SALURBAL. Glob Challenges. 2019;3(4):1800013. 10.1002/gch2.201800013 PMC6450446 31565372

[6] United Nations . World Population Prospects—Population Division—United Nations. World population prospects. https://population.un.org/wpp/DataQuery/. Published 2019. Accessed July 27, 2020.

[7] Kepper MM, Myers CA, Denstel KD, Hunter RF, Guan W, Broyles ST. The neighborhood social environment and physical activity: a systematic scoping review. Int J Behav Nutr Phys Act. 2019;16(1):1‐16. 10.1186/s12966-019-0873-7 31815626 PMC6902518

[8] Hunter RF, Ball K, Sarmiento OL. Socially awkward: how can we better promote walking as a social behaviour? Br J Sports Med. 2018;52(12):757‐758. 10.1136/bjsports-2017-098564 29475837

[9] Kumanyika S, Taylor WC, Grier SA, et al. Community energy balance: a framework for contextualizing cultural influences on high risk of obesity in ethnic minority populations. Prev Med (Baltim). 2012;55(5):371‐381. 10.1016/j.ypmed.2012.07.002 22800683

[10] Timperio A, Reid J, Veitch J. Playability: built and social environment features that promote physical activity within children. Curr Obes Rep. 2015;4(4):460‐476. 10.1007/s13679-015-0178-3 26399255

[11] Marzi I, Reimers AK. Children's independent mobility: current knowledge, future directions, and public health implications. Int J Environ Res Public Health. 2018;15(11). 10.3390/ijerph15112441 PMC6267483 30388880

[12] National Institutes of Health . Childhood Obesity Prevention Across Borders: The Promise of US‐Latin American Research Collaboration. Fogarty International Center. https://www.fic.nih.gov/About/center‐global‐health‐studies/Pages/childhood‐obesity‐prevention‐across‐borders.aspx. Published 2020. Accessed November 25, 2020.

[13] Spence JC, Lee RE. Toward a comprehensive model of physical activity. Psychol Sport Exerc. 2003;4(1):7‐24. 10.1016/S1469-0292(02)00014-6

[14] Sallis JF, Bull F, Guthold R, et al. Progress in physical activity over the Olympic quadrennium. Lancet. 2016;388(10051):1325‐1336. 10.1016/S0140-6736(16)30581-5 27475270

[15] Smith M, Hosking J, Woodward A, et al. Systematic literature review of built environment effects on physical activity and active transport—an update and new findings on health equity. Int J Behav Nutr Phys Act. 2017;14(1):1‐28. 10.1186/s12966-017-0613-9 29145884 PMC5693449

[16] Masoumi HE. Associations of built environment and children's physical activity: a narrative review. Rev Environ Health. 2017;32(4):315‐331. 10.1515/reveh-2016-0046 28809754

[17] D'Haese S, Vanwolleghem G, Hinckson E, et al. Cross‐continental comparison of the association between the physical environment and active transportation in children: a systematic review. Int J Behav Nutr Phys Act. 2015;12(1):145. 10.1186/s12966-015-0308-z 26610344 PMC4660808

[18] Jones SA, Moore LV, Moore K, et al. Disparities in physical activity resource availability in six US regions. Prev Med (Baltim). 2015;78:17‐22. 10.1016/j.ypmed.2015.05.028 PMC4547867 26067479

[19] Cerin E, Baranowski T, Barnett A, et al. Places where preschoolers are (in)active: an observational study on Latino preschoolers and their parents using objective measures. Int J Behav Nutr Phys Act. 2016;13(1):1‐13. 10.1186/s12966-016-0355-0 26928561 PMC4772489

[20] Ross SET, Francis LA. Physical activity perceptions, context, barriers, and facilitators from a Hispanic child's perspective. Int J Qual Stud Health Well‐Being. 2016;11(1):1‐11. 10.3402/qhw.v11.31949 PMC4989179 27534946

[21] Grzywacz JG, Arcury TA, Trejo G, Quandt SA. Latino mothers in farmworker families' beliefs about preschool children's physical activity and play. J Immigr Minor Health. 2016;18(1):234‐242. 10.1007/s10903-014-9990-1 24522435 PMC4133327

[22] Umstattd Meyer MR, Sharkey JR, Patterson MS, Dean WR. Understanding contextual barriers, supports, and opportunities for physical activity among Mexican‐origin children in Texas border colonias: a descriptive study. BMC Public Health. 2013;13(1):14. 10.1186/1471-2458-13-14 23297793 PMC3558355

[23] Carroll‐Scott A, Gilstad‐Hayden K, Rosenthal L, et al. Disentangling neighborhood contextual associations with child body mass index, diet, and physical activity: the role of built, socioeconomic, and social environments. Soc Sci Med. 2013;95:106‐114. 10.1016/j.socscimed.2013.04.003 23642646 PMC4058500

[24] Lavin Fueyo J, Totaro Garcia LM, Mamondi V, Pereira Alencar G, Florindo AA, Berra S. Neighborhood and family perceived environments associated with children's physical activity and body mass index. Prev Med. 2016;82:35‐41. 10.1016/j.ypmed.2015.11.005 26582209

[25] Jauregui A, Soltero E, Santos‐Luna R, et al. A multisite study of environmental correlates of active commuting to school in Mexican children. J Phys Act Health. 2016;13(3):325‐332. 10.1123/jpah.2014-0483 26284941

[26] Dias AF, Gaya AR, Pizarro AN, et al. Perceived and objective measures of neighborhood environment: Association with active commuting to school by socioeconomic status in Brazilian adolescents. J Transp Health. 2019;14:e100612. 10.1016/j.jth.2019.100612

[27] Lee RE, Soltero EG, Jáuregui A, et al. Disentangling associations of neighborhood street scale elements with physical activity in Mexican school children. Environ Behav. 2016;48(1):150‐171. 10.1177/0013916515615389

[28] Melo E, Barros M, Siqueira R, Hino AA, Meneses C, Cazuza J. Is the environment near school associated with active commuting to school among preschoolers? Brazilian J Kinanthropometry Hum Perform. 2013;15(4):393‐404. 10.5007/1980-0037.2013v15n4p393

[29] McDonald NC. Active transportation to school. Trends among U.S. schoolchildren, 1969–2001. Am J Prev Med. 2007;32(6):509‐516. 10.1016/j.amepre.2007.02.022 17533067

[30] Kontou E, McDonald NC, Brookshire K, Pullen‐Seufert NC, La Jeunesse S. U.S. active school travel in 2017: prevalence and correlates. Prev Med Rep. 2020;17:101024. 10.1016/j.pmedr.2019.101024 31921574 PMC6948264

[31] García I, Keuntae K. Active commute to school, physical activity and health of Hispanic high school in students in the United States. In: Oviedo D, Villamizar N, Ardila A, eds. Urban Mobility and Social Equity in Latin America: Evidence, Concepts, Methods. Vol 12. Transport and Sustainability. Emerald Publishing Limited; 2020:149‐168. 10.1108/S2044-99412020000012011.

[32] Arango CM, Parra DC, Eyler A, et al. Walking or bicycling to school and weight status among adolescents from Montería, Colombia. J Phys Act Health. 2011;8(s2):S171‐S177. 10.1123/jpah.8.s2.s171 28829700

[33] Jáuregui A, Medina C, Salvo D, Barquera S, Rivera‐Dommarco JA. Active commuting to school in Mexican adolescents: evidence from the Mexican National Nutrition and Health Survey. J Phys Act Health. 2015;12(8):1088‐1095. 10.1123/jpah.2014-0103 25247894

[34] Rodríguez‐Rodríguez F, Cristi‐Montero C, Celis‐Morales C, Escobar‐Gómez D, Chillón P. Impact of distance on mode of active commuting in Chilean children and adolescents. Int J Environ Res Public Health. 2017;14(11):1‐9. 10.3390/ijerph14111334 PMC5707973 29099044

[35] Becker L, Fermino R, Lima A, Rech C, Añez C, Reis R. Perceived barriers for active commuting to school among adolescents from Curitiba, Brazil. Rev Bras Atividade Física Saúde. 2017;22(1):24‐34. 10.12820/rbafs.v.22n1p24-34

[36] González SA, Sarmiento OL, Lemoine PD, et al. Active school transport among children from Canada, Colombia, Finland, South Africa, and the United States: a tale of two journeys. Int J Environ Res Public Health. 2020;17(11):3847. 10.3390/ijerph17113847 PMC7312928 32481728

[37] Sullivan SM, Broyles ST, Barreira TV, et al. Associations of neighborhood social environment attributes and physical activity among 9–11 year old children from 12 countries. Health Place. 2017;46(May):183‐191. 10.1016/j.healthplace.2017.05.013 28544991

[38] Hermosillo‐Gallardo ME, Sebire SJ, Jago R. Perception of safety and its association with physical activity in adolescents in Mexico. Am J Prev Med. 2020;58(5):748‐755. 10.1016/j.amepre.2019.12.007 32063387

[39] Lovasi GS, Jacobson JS, Quinn JW, Neckerman KM, Ashby‐Thompson MN, Rundle A. Is the environment near home and school associated with physical activity and adiposity of urban preschool children? J Urban Health. 2011;88(6):1143‐1157. 10.1007/s11524-011-9604-3 21826583 PMC3232416

[40] Mier N, Lee C, Smith ML, et al. Mexican‐American children's perspectives: neighborhood characteristics and physical activity in Texas‐Mexico border colonias. J Environ Health. 2013;76(3):8‐16. 24288846

[41] Olvera N, Smith DW, Lee C, et al. Hispanic maternal and children's perceptions of neighborhood safety related to walking and cycling. Health Place. 2012;18(1):71‐75. 10.1016/j.healthplace.2011.08.022 22243908

[42] Lindsay AC, Sussner K, Greaney M, Peterson K. Influence of social context on eating, physical activity, and sedentary behaviors of Latina mothers and their preschool‐age children. Health Educ Behav. 2009;36(1):81‐96. 10.1177/1090198107308375 18689491 PMC3089594

[43] Alex Quistberg D, Diez Roux AV, Bilal U, et al. Building a data platform for cross‐country urban health studies: the SALURBAL study. J Urban Health. 96(2):311‐337. 10.1007/s11524-018-00326-0 PMC6458229 30465261

[44] Boeing G. OSMNX: new methods for acquiring, constructing, analyzing, and visualizing complex street networks. Comput Environ Urban Syst. 2017;65:126‐139. 10.1016/j.compenvurbsys.2017.05.004

[45] Lee RE, Mama SK, Adamus‐Leach HJ, Soltero EG. Contribution of neighborhood income and access to quality physical activity resources to physical activity in ethnic minority women over time. Am J Health Promot. 2015;29(4):210‐216. 10.4278/ajhp.130403-QUAN-148 24524382 PMC4515360

[46] Hunter R, Cleary A, Cleland C, Braubach M. Urban green space interventions and health: a review of impacts and effectiveness. World Health Organization; 2017. https://www.euro.who.int/en/health-topics/environment-and‐health/urban‐health/publications/2017/urban‐green‐space‐interventions‐and‐health‐a‐review‐of‐impacts‐and‐effectiveness.‐full‐report‐2017. Accessed November 25, 2020.

[47] Lee RE, Booth KM, Reese‐Smith JY, Regan G, Howard HH. The Physical Activity Resource Assessment (PARA) instrument: evaluating features, amenities and incivilities of physical activity resources in urban neighborhoods. Int J Behav Nutr Phys Act. 2005;2(1):1‐9. 10.1186/1479-5868-2-13 16162285 PMC1262748

[48] Engelberg JK, Conway TL, Geremia C, et al. Socioeconomic and race/ethnic disparities in observed park quality. BMC Public Health. 2016;16(395):2‐11. 10.1186/s12889-016-3055-4 27176854 PMC4866396

[49] de Moraes Ferrari GL, Kovalskys I, Fisberg M, et al. Socio‐demographic patterns of public, private and active travel in Latin America: cross‐sectional findings from the ELANS study. J Transp Health. 2020;16:100788. 10.1016/j.jth.2019.100788

[50] McCray TM, Mora S. Analyzing the activity spaces of low‐income teenagers: how do they perceive the spaces where activities are carried out? J Urban Aff. 2011;33(5):511‐528. 10.1111/j.1467-9906.2011.00563.x

[51] Hocking B, Sturgeon B, Dixon J, et al. Place‐identity and urban policy: sharing leisure spaces in the ‘post‐conflict’ city. In: Discourses of Identity in Liminal Places and Spaces; 2019:166‐192. 10.4324/9781351183383-8.

[52] Dixon J, Tredoux C, Davies G, et al. Parallel lives: intergroup contact, threat, and the segregation of everyday activity spaces. J Pers Soc Psychol. 2019;118(3):457‐480. 10.1037/pspi0000191 31045387

[53] Davies G, Dixon J, Tredoux CG, et al. Networks of (dis)connection: mobility practices, tertiary streets, and sectarian divisions in North Belfast. Ann am Assoc Geogr. 2019;109(6):1729‐1747. 10.1080/24694452.2019.1593817

[54] Alberico CO, Schipperijn J, Reis RS. Use of global positioning system for physical activity research in youth: ESPAÇOS Adolescentes, Brazil. Prev Med (Baltim). 2017;103S:S59‐S65. 10.1016/j.ypmed.2016.12.026 PMC5481504 28024861

[55] Camargo EM, de Alberico CO, Lopes AAS, Schipperijn J, Reis RS. Characteristics of the built environment on GPS‐determined bicycle routes used by adolescents. Rev Bras Atividade Física Saúde. 2019;24:1‐7. 10.12820/rbafs.24e0106

[56] Soltero EG, Cerin E, Lee RE, O'Connor TM. Associations between objective and self‐report measures of traffic and crime safety in Latino parents of preschool children. J Immigr Minor Health. 2017;19(5):1109‐1120. 10.1007/s10903-016-0498-8 27680746

[57] Larrea I, Muela A, Miranda N, Barandiaran A. Children's social play and affordance availability in preschool outdoor environments. Eur Early Child Educ Res J. 2019;27(2):185‐194. 10.1080/1350293X.2019.1579546

[58] Spencer C, Woolley H. Children and the city: a summary of recent environmental psychology research. Child Care Health Dev. 2000;26(3):181‐198. 10.1046/j.1365-2214.2000.00125.x 10921437

[59] Montgomery SC, Donnelly M, Bhatnagar P, Carlin A, Kee F, Hunter RF. Peer social network processes and adolescent health behaviors: a systematic review. Prev Med (Baltim). 2020;130:105900. 10.1016/j.ypmed.2019.105900 31733224

[60] King AC. Theory's role in shaping behavioral health research for population health. Int J Behav Nutr Phys Act. 2015;12:1‐5. 26612691 10.1186/s12966-015-0307-0 PMC4660825

[61] Díaz del Castillo A, Sarmiento OL, Reis RS, Brownson RC. Translating evidence to policy: urban interventions and physical activity promotion in Bogotá, Colombia and Curitiba, Brazil. Transl Behav Med. 2011;1(2):350‐360. 10.1007/s13142-011-0038-y 24073055 PMC3717654

[62] Valente TW. Network interventions. Science (80‐). 2012;336(6090):49‐53. 10.1126/science.1217330 22767921

[63] Hunter RF, De La Haye K, Murray JM, et al. Social network interventions for health behaviours and outcomes: a systematic review and meta‐analysis. PLoS Med. 2019;16(9):e1002890. 10.1371/journal.pmed.1002890 31479454 PMC6719831

[64] Murray JM, Kimbrough EO, Krupka EL, et al. Confirmatory factor analysis comparing incentivized experiments with self‐report methods to elicit adolescent smoking and vaping social norms. Sci Rep. 2020;10(1):15818. 10.1038/s41598-020-72784-z 32978471 PMC7519107

[65] Hunter RF, Montes F, Murray JM, et al. MECHANISMS study: using game theory to assess the effects of social norms and social networks on adolescent smoking in schools—study protocol. Front Public Health. 2020;8(377):1‐9. 10.3389/fpubh.2020.00377 32850598 PMC7417659

[66] Corepal R, Best P, O'Neill R, et al. Exploring the use of a gamified intervention for encouraging physical activity in adolescents: a qualitative longitudinal study in Northern Ireland. BMJ Open. 2018;8(4):e019663. 10.1136/bmjopen-2017-019663 PMC5914771 29678971

[67] Simpkins SD, Schaefer DR, Price CD, Vest AE. Adolescent friendships, BMI, and physical activity: untangling selection and influence through longitudinal social network analysis. J Res Adolesc. 2013;23(3):537‐549. 10.1111/j.1532-7795.2012.00836.x PMC3821778 24222971

[68] Zhang J, Shoham DA, Tesdahl E, Gesell SB. Network interventions on physical activity in an afterschool program: an agent‐based social network study. Am J Public Health. 2015;105(S2):S236‐S243. 10.2105/AJPH.2014.302277 25689202 PMC4355707

[69] Guerra AM, Montes F, Useche AF, et al. Effects of a physical activity program potentiated with ICTs on the formation and dissolution of friendship networks of children in a middle‐income country. Int J Environ Res Public Health. 2020;17(16):1‐21. 10.3390/ijerph17165796 PMC7459696 32796502

[70] King AC, Winter SJ, Sheats JL, et al. Leveraging citizen science and information technology for population physical activity promotion. Transl J Am Coll Sport Med. 2016;1(4):30‐44. 10.1249/TJX.0000000000000003 PMC4978140 27525309

[71] King AC, Winter SJ, Chrisinger BW, Hua J, Banchoff AW. Maximizing the promise of citizen science to advance health and prevent disease. Prev Med (Baltim). 2019;119:44‐47. 10.1016/j.ypmed.2018.12.016 PMC6687391 30593793

[72] Buman MP, Winter SJ, Sheats JL, et al. The stanford healthy neighborhood discovery tool: a computerized tool to assess active living environments. Am J Prev Med. 2013;44(4):e41‐e47. 10.1016/j.amepre.2012.11.028 23498112 PMC3601583

[73] Rodriguez NM, Arce A, Kawaguchi A, et al. Enhancing safe routes to school programs through community‐engaged citizen science: two pilot investigations in lower density areas of Santa Clara County, California, USA. BMC Public Health. 2019;19(1):1‐12. 10.1186/s12889-019-6563-1 30823917 PMC6397479

[74] Rosas LG, Salvo D, Winter SJ, et al. Harnessing technology and citizen science to support neighborhoods that promote active living in Mexico. J Urban Health. 2016;93(6):953‐973. 10.1007/s11524-016-0081-6 27752825 PMC5126018

[75] King AC, Winter SJ, Chrisinger BW, Hua J, Banchoff AW. Maximizing the promise of citizen science to advance health and prevent disease. Prev Med. 2019;119(December 2018):44‐47. 30593793 10.1016/j.ypmed.2018.12.016 PMC6687391

[76] King AC, Odunitan‐wayas FA, Chaudhury M, et al. Community‐based approaches to reducing health inequities and fostering environmental justice through global youth‐engaged citizen science. Int J Environm Res Public Heal. 2021;18:892(892):1‐29. 10.3390/ijerph18030892 PMC7908382 33494135

[77] Winter SJ, Goldman Rosas L, Padilla Romero P, et al. Using citizen scientists to gather, analyze, and disseminate information about neighborhood features that affect active living. J Immigr Minor Health. 2016;18(5):1126‐1138. 10.1007/s10903-015-0241-x 26184398 PMC4715987

[78] Rubio MA, Triana C, King AC, et al. Engaging citizen scientists to build healthy park environments in Colombia. Health Promot Int. 2020;35:daaa031. 10.1093/heapro/daaa031 PMC7954214 32361761

[79] González SA, Rubio MA, Triana CA, King AC, Banchoff AW, Sarmiento OL. Building healthy schools through technology‐enabled citizen science: the case of the our voice participatory action model in schools from Bogotá, Colombia. Glob Public Health. 2021;1‐17. 10.1080/17441692.2020.1869285 33427068

[80] Montes F, Sarmiento OL, Rodríguez AL, et al. Physical inactivity and substance use in rural areas: socially transmitted conditions? In: Latin American Conference on Complex Networks. Cartagena; 2019.

[81] Umstattd Meyer MR, Bridges CN, Schmid TL, Hecht AA, Pollack Porter KM. Systematic review of how play streets impact opportunities for active play, physical activity, neighborhoods, and communities. BMC Public Health. 2019;19(1):1‐17. 10.1186/s12889-019-6609-4 30902073 PMC6431069

[82] Ziviani J, Wadley D, Ward H, Macdonald D, Jenkins D, Rodger S. A place to play: socioeconomic and spatial factors in children's physical activity. Aust Occup Ther J. 2008;55(1):2‐11. 10.1111/j.1440-1630.2006.00646.x 20887428

[83] Cortinez‐O'Ryan A, Albagli A, Sadarangani KP, Aguilar‐Farias N. Reclaiming streets for outdoor play: a process and impact evaluation of “Juega en tu Barrio” (play in your neighborhood), an intervention to increase physical activity and opportunities for play. PLoS One. 2017;12(7):e0180172. 10.1371/journal.pone.0180172 28671984 PMC5495338

[84] Morton KL, Atkin AJ, Corder K, Suhrcke M, van Sluijs EMF. The school environment and adolescent physical activity and sedentary behaviour: a mixed‐studies systematic review. Obes Rev. 2016;17(2):142‐158. 10.1111/obr.12352 26680609 PMC4914929

[85] Sallis JF, Floyd MF, Rodríguez DA, Saelens BE. Role of built environments in physical activity, obesity, and cardiovascular disease. Circulation. 2012;125(5):729‐737. 10.1161/CIRCULATIONAHA.110.969022 22311885 PMC3315587

[86] Urban Impact Lab, ConnectFamilias, Miami Children's Initiative, The Children's Trust. *How to Do a Play Street*. Miami; 2019. 10.4324/9781315175423-3

[87] Daude C, Fajardo G, Brassiolo P, et al. Crecimiento Urbano y Acceso a Oportunidades: Un Desafío Para América Latina RESUMEN EJECUTIVO. CAF; 2017. https://scioteca.caf.com/handle/123456789/1090. Accessed November 30, 2020.

[88] Alcaldía Mayor de Bogotá. *Niños Primero. Movilidad Escolar Segura Para La Felicidad de Las Niñas y Los Niños de Bogotá*. Bogotá; 2019.

[89] DiMaggio C, Frangos S, Li G. National safe routes to school program and risk of school‐age pedestrian and bicyclist injury. Ann Epidemiol. 2016;26(6):412‐417. 10.1016/j.annepidem.2016.04.002 27230492 PMC5248654

[90] McDonald NC, Barth PH, Steiner RL. Assessing the distribution of safe routes to school program funds, 2005–2012. Am J Prev Med. 2013;45(4):401‐406. 10.1016/j.amepre.2013.04.024 24050415

[91] Heath EM, Coleman KJ. Evaluation of the Institutionalization of the Coordinated Approach to Child Health (CATCH) in a U.S./Mexico border community. Health Educ Behav. 2002;29(4):444‐460. 10.1177/109019810202900405 12137238

[92] Hoelscher DM, Springer A, Menendez TH, Cribb PW, Kelder SH. From NIH to Texas schools: policy impact of the Coordinated Approach to Child Health (CATCH) program in Texas. J Phys Act Health. 2011;8(Suppl 1):S5‐S7. 10.1123/jpah.8.s1.s5 21350263

[93] Reis RS, Salvo D, Ogilvie D, Lambert EV, Goenka S, Brownson RC. Scaling up physical activity interventions worldwide: stepping up to larger and smarter approaches to get people moving. Lancet. 2016;388(10051):1337‐1348. 10.1016/S0140-6736(16)30728-0 27475273 PMC5193005

[94] Instituto Nacional de Salud Pública . “Vida Saludable”, nueva materia en planes de estudio. Gobierno de México. https://insp.mx/avisos/vida‐saludable‐nueva‐materia‐en‐planes‐de‐estudio. Published 2020. Accessed December 22, 2020.

[95] Secretaria de Salud, Secretaría de Educación Pública. *Salud Escolar: Escuelas Saludables y Activas*. Mexico City; 2019.

[96] Zieff SG, Chaudhuri A, Musselman E. Creating neighborhood recreational space for youth and children in the urban environment: play (ing in the) streets in San Francisco. Child Youth Serv Rev. 2016;70:95‐101. 10.1016/j.childyouth.2016.09.014

[97] Hindhede AL, Aagaard‐Hansen J. Using social network analysis as a method to assess and strengthen participation in health promotion programs in vulnerable areas. Health Promot Pract. 2017;18(2):175‐183. 10.1177/1524839916686029 28118745

